# Orthokeratinized Odontogenic Cyst in the Posterior Maxilla and Its Surgical Management: A Report of a Rare Case

**DOI:** 10.7759/cureus.78052

**Published:** 2025-01-27

**Authors:** A. Saneem Ahamed, C. Chandoorya, A. Venkata Maheedhar, Sree Ram Subba Reddy Gudimetla, Kapil Dev Kumar

**Affiliations:** 1 Oral and Maxillofacial Surgery, Priyadarshini Dental College and Hospital, Pandur, IND

**Keywords:** enucleation, intraosseous cyst, kcot, maxilla, odontogenic keratocyst, orthokeratinized odontogenic cyst, unilocular radiolucency

## Abstract

Orthokeratinized odontogenic cyst (OOC) is a rare developmental anomaly of the jaw, now formally recognized as a distinct entity due to its unique biological characteristics, specialized histopathological features, reduced aggressiveness, and significantly lower recurrence rate compared to odontogenic keratocysts. This report describes a case in which the cyst was surgically enucleated, and a comprehensive histopathological evaluation of the specimen confirmed the diagnosis of OOC. The report provides an in-depth exploration of the clinical management and therapeutic strategies employed in addressing this atypical and clinically significant pathological condition.

## Introduction

An orthokeratinized odontogenic cyst (OOC) is an infrequent intraosseous cyst identified by its orthokeratinized epithelial lining and marked clinical indolence. Initially delineated by Schultz in 1927 as a variant of odontogenic keratocysts [1], it is presently classified as a keratocystic odontogenic tumor (KCOT) [2]. An OOC is characterized by distinctive histopathological attributes and clinical tendencies. Its histogenesis is believed to arise either from the vestiges of the dental lamina or the basal cell layer of the oral mucosal epithelium [3].

Empirical data suggests that male individuals exhibited a higher predilection than females, commonly manifesting this lesion during the third and fourth decades [4]. The mandible is affected twice as frequently as the maxilla [5]. Radiographically, the cyst manifests as a precisely demarcated, either solitary or multilocular radiolucent lesion, occasionally exhibiting an association with either an impacted tooth or the root, without inducing resorption [6].

The selection of treatment is contingent upon the cyst's dimensions, recurrence risk status, and the radiographic documentation of cortical perforation [7]. The recurrence rate of OOC is notably low, obviating the necessity for invasive therapeutic modalities. Conservative methods suffice for the management of OOC [8].

This report endeavors to explicate the diagnostic criteria, surgical intricacies, and resultant outcomes inherent to OOCs. Intriguingly, the case reported involves a female patient with an OOC in the maxilla.

## Case presentation

A 34-year-old female patient presented to the Department of Oral and Maxillofacial Surgery with the chief complaint of recurrent episodes of dull and intermittent pain in the upper right posterior tooth region, persisting over the past four months. The patient's medical history revealed that she had a preexisting physical disability due to polio. Moreover, she had been undergoing psychiatric treatment for the preceding two months. Notably, the patient also disclosed a history of trauma that occurred four years prior.

**Figure 1:**
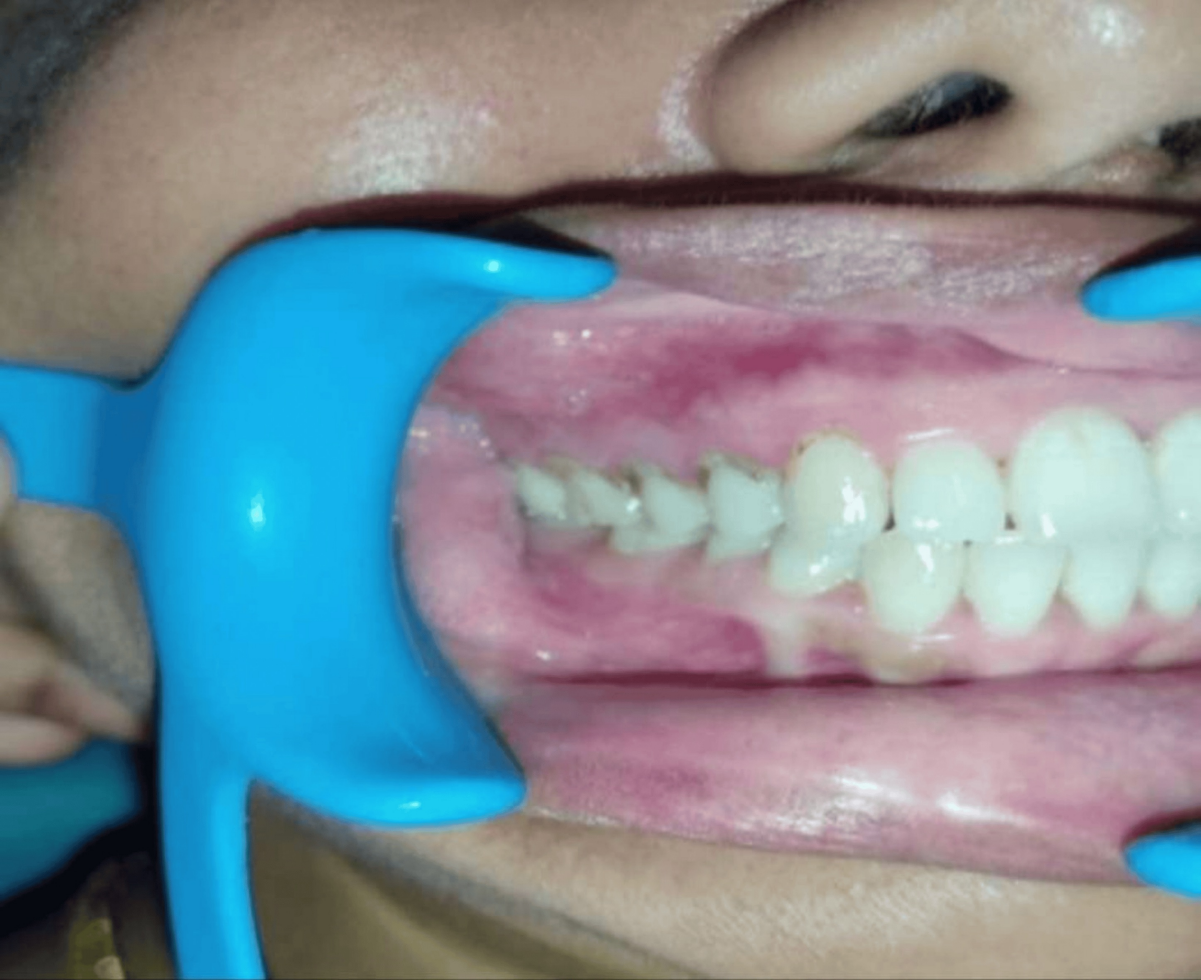
Intraoral examination reveals a 2x3 cm swelling in the right posterior region of the maxilla.

Upon intraoral examination, a firm and tender swelling measuring approximately 2x3 cm was detected in the alveolar mucosa of the maxillary right posterior region (Figure 1).

**Figure 2:**
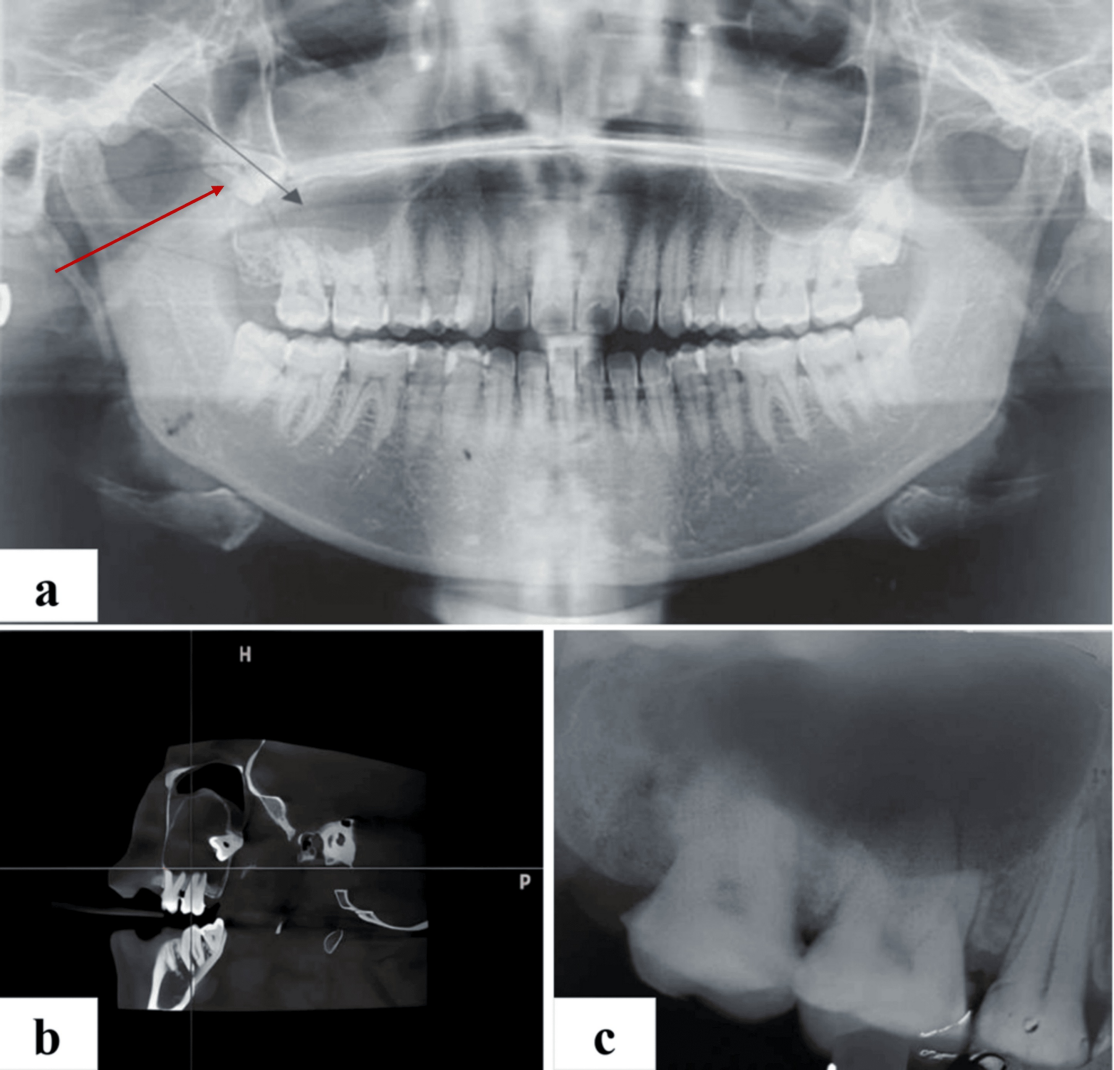
(a) Preoperative orthopantomograph (OPG) showing unilocular well defined radiolucency in maxillary posterior tooth region. (b) CBCT showing unilocular lesion in right posterior maxilla. (c) Intraoral periapical radiograph showing well-defined radiolucency. CVCT: cone-beam computed tomography

The patient exhibited mild to moderate tenderness in the zygomaticomaxillary buttress. Orthopantomography (OPG) revealed a lesion in the maxillary right posterior tooth region. It presented as a well-defined, unilocular radiolucency (Figure 2a). The cyst's periphery was well-defined and corticated, and it was associated with the cemento-enamel junction in relation to tooth 18. The maxillary sinus exhibited displacement due to the impaction of teeth 18 and 28. 

Following cone-beam computed tomography (CBCT) analysis, a unilocular, low-density lesion became apparent in the right posterior maxilla (Figure 2b), measuring approximately 13.7 mm buccolingually, 24.3 mm anteroposteriorly, and 31.0 mm superoinferiorly in its greatest dimension. The lesion exhibited substantial biocritical expansion. Furthermore, a hyperdense, tooth-like structure was identified within the lesion, with the impacted tooth situated approximately 11.8 mm above the alveolus of the maxilla in relation to tooth 18. The impacted tooth was firmly adhered to the distal wall of the maxillary sinus. Notable findings encompassed opacification of the right maxillary sinus with cortical perforation. Importantly, there was no indication of root resorption. In light of the orthopantomogram and CBCT analysis, the findings strongly suggested the presence of a dentigerous cyst. 

The intraoral periapical radiograph (IOPA) revealed that the lesion extended from the right second premolar to the right second molar (Figure 2c), with the presence of an impacted third molar. Furthermore, there was a well-defined periapical radiolucency noted in relation to teeth 15-17.

**Figure 3:**
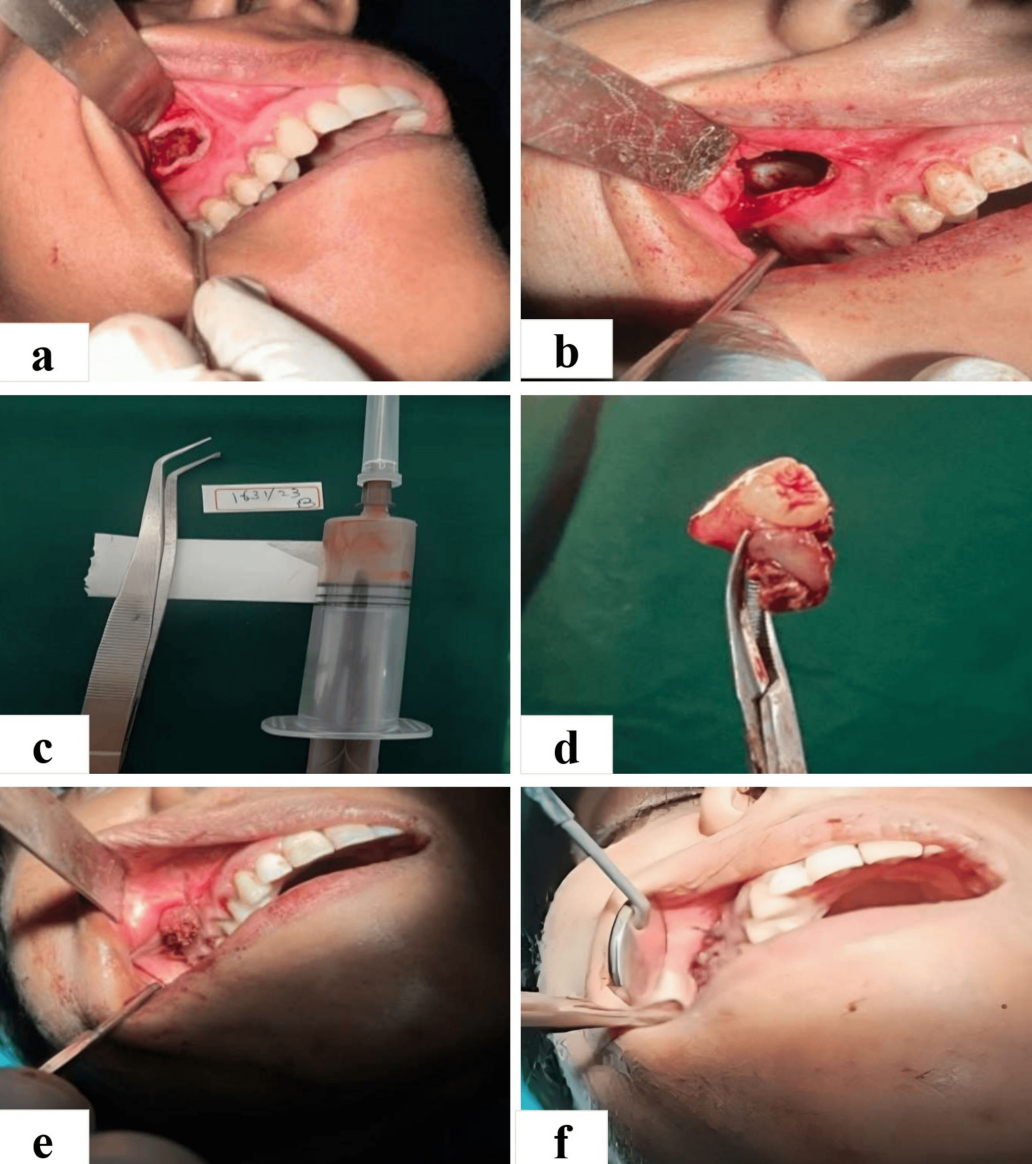
(a) Elevated mucoperiosteal flap following a vestibular incision. (b) Cystic cavity after creating a bony window. (c) FNAC showing straw-coloured fluid intermixed with blood. (d) Impacted tooth associated with cyst. (e) Tincture benzoin with roller gauze placed inside the cavity. (f) Flap closure with a simple interrupted suture. FNAC: fine needle aspiration cytology

The patient was scheduled for the enucleation of the cyst under local anesthesia, and written informed consent was duly obtained. The planned procedure was performed under local anesthesia. The treatment involved the enucleation process, which commenced with a vestibular incision made in relation to teeth 13-17, and a mucoperiosteal flap was raised (Figure 3a). A window opening was created on the lateral wall of the maxillary sinus in relation to teeth 13-17, measuring 2 cm × 1 cm (Figure 3b). The cystic content was aspirated using an 18-gauge needle, revealing a serosanguinous fluid, after which the cystic lining was meticulously removed and detached from the bone (Figure 3c). The impacted tooth was extracted from the cavity (Figure 3d). Tincture benzoin, along with roller gauze, was applied (Figure 3e), and a simple interrupted suture was placed using 3-0 Vicryl, with the end of the roller gauze left in place to facilitate the process (Figure 3f).

Hemostasis was successfully achieved, and medications were prescribed to the patient for postoperative care. The packing was replaced during the weekly recall visits for three months following the initial surgery. Immediately after the enucleation, the specimen was sent for histopathological examination. The histopathological diagnosis indicated the presence of an OOC with evidence of inflammation.

## Discussion

An OOC is an infrequent and developmental odontogenic cyst originating from the dental lamina [9]. In 1981, Wright coined the term "orthokeratinized variant of OKC," delineating it as a distinct clinical entity [10]. It was not until 1998 that Li et al. proposed the term OOC, which has since become the widely accepted nomenclature [11]. OOCs comprise 7-17% of all keratinizing jaw cysts [12]. OOCs are usually asymptomatic, and slow-growing swelling is the most frequent presenting symptom, occasionally accompanied by pain [13]. In our case, the primary concern was recurrent episodes of dull and intermittent pain. Radiographically, OOCs appear as radiolucent solitary lesions which are frequently associated with impacted teeth [5].

**Figure 4:**
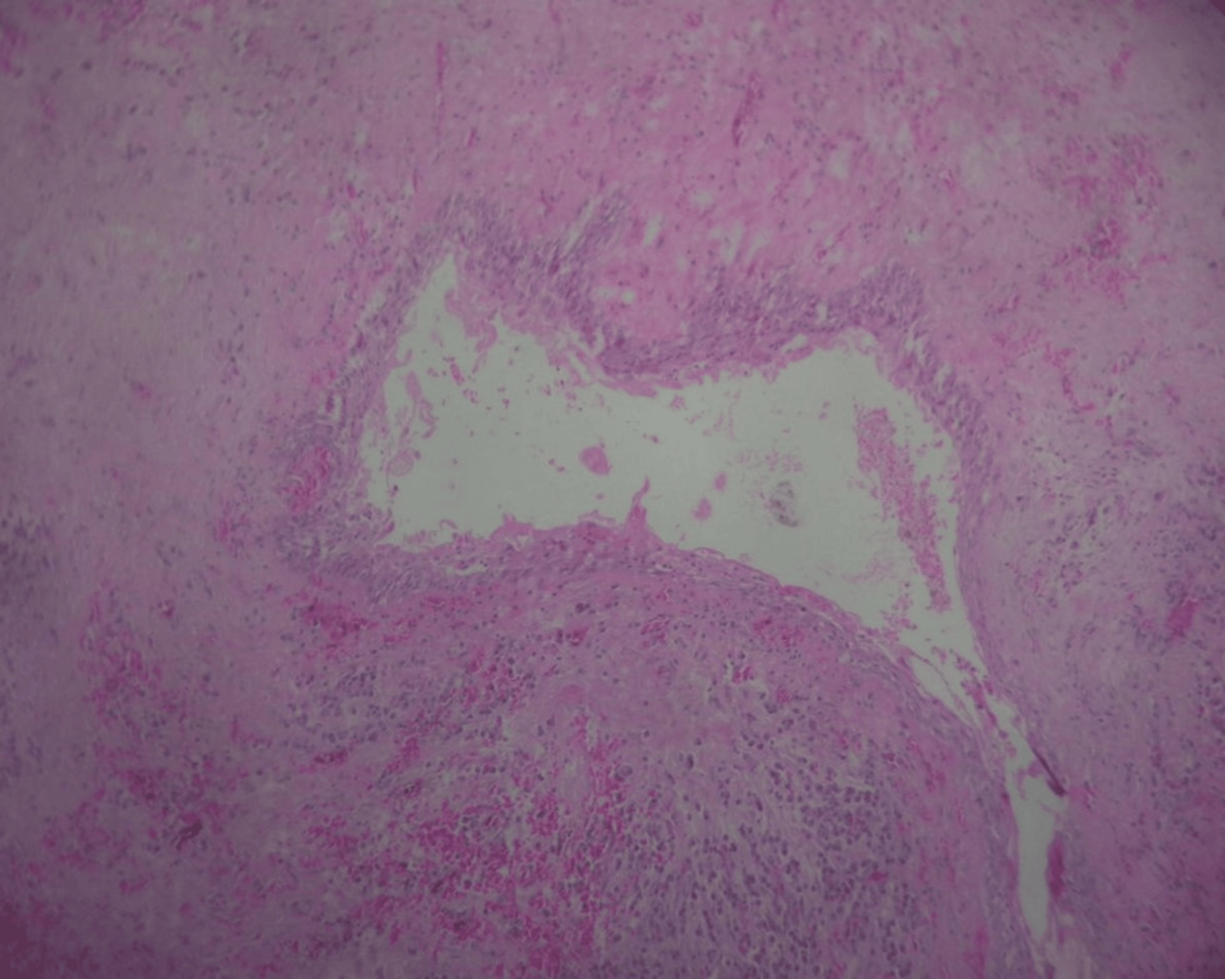
Histopathology reveals a lining epithelium that is orthokeratinized and the basal layer exhibits cuboidal cells, suggestive of orthokeratinized odontogenic cyst

Histologically, OOC is characterized by a consistently stratified squamous epithelium, ranging from four to nine layers in thickness [13]. In the present case, the histological examination revealed a lining epithelium that was orthokeratinized, exhibiting uniform thickness spanning three to eight layers; the basal layer exhibited palisade cuboidal or flat cells displaying nuclear hyperchromatism (Figure 4).

The intermediate layer comprises polyhedral cells with eosinophilic cytoplasm and a prominent superficial layer of orthokeratin. In contrast, the keratocystic odontogenic tumor (KCOT) displays an epithelium spanning 5-10 layers, with basal cells featuring elongated nuclei and a distinctive superficial corrugated layer of parakeratin. The comprehensive differential diagnosis of OOC encompasses various radiolucent lesions within the jaws, primarily odontogenic entities such as dentigerous cysts or paradental cysts. In addition to these, consideration should be given to odontogenic tumors, including ameloblastoma and KCOT. Notably, OOC exhibits analogous radiographic features to both ameloblastoma and KCOT [2,14].

Two universally acknowledged approaches for treating OKC and OOC are resection or enucleation coupled with peripheral ostectomy and decompression [4]. In the current case, the therapeutic course for OOC involved enucleation, ensuring complete excision of the cyst, coupled with the surgical extraction of impacted teeth. Due to the low recurrence of OOC, its treatment plan is conservative and enucleation is the recommended method.

## Conclusions

OOCs present a rare yet potentially malignant condition that demands careful management by oral and maxillofacial surgeons. Differentiating OOC from other lesions, particularly odontogenic keratocysts (OKC), is crucial due to differences in treatment and prognosis. Diagnosis remains challenging, requiring a thorough comparison of radiolucent jaw lesions. Treatment typically involves enucleation with curettage, offering an excellent prognosis with low recurrence rates compared to other lesions. Overall, precise diagnosis and tailored surgical intervention are key to ensuring optimal outcomes in managing OOC.
